# Complete Coding Sequence of a Lumpy Skin Disease Virus from an Outbreak in Bulgaria in 2016

**DOI:** 10.1128/MRA.00977-20

**Published:** 2020-10-22

**Authors:** Elisabeth Mathijs, Frank Vandenbussche, Emiliya Ivanova, Andy Haegeman, Laetitia Aerts, Ilse De Leeuw, Steven Van Borm, Kris De Clercq

**Affiliations:** a Sciensano, EURL Capripox, Unit Exotic Viruses and Particular Diseases, Ukkel, Belgium; b National Diagnostic and Research Veterinary Medical Institute, Sofia, Bulgaria; KU Leuven

## Abstract

Lumpy skin disease (LSD) is an emerging cattle disease with serious economic consequences. We report the complete coding sequence of LSD virus 210LSD-249/BUL/16, detected in a blood sample from a diseased cow during an outbreak in Bulgaria (Kabile Village, Yambol Region) in June 2016.

## ANNOUNCEMENT

Lumpy skin disease (LSD) is a major disease in cattle caused by lumpy skin disease virus (LSDV), which belongs to the genus *Capripoxvirus* in the family *Poxviridae*. The disease, no longer limited to Africa, spread to the Middle East in 2012 and Turkey in 2013. Further expanding northwestward, it quickly spread across the Balkans in 2016 (1), with a total of 217 LSD outbreaks reported in Bulgaria, where the disease was controlled by vaccination by the next year (2). We report the complete coding sequence of an LSDV from an outbreak in the Yambol Region of Bulgaria in June 2016 (210LSD-249/BUL/16).

DNA was extracted from a blood sample from a cow with clinical signs using the Puregene core kit A (Qiagen) as previously described (3). Presequencing enrichment relied on the amplification of 23 overlapping PCR products (ranging between 7,417 and 7,852 bp) covering the entire genome, using Q5 high-fidelity DNA polymerase (New England Biolabs), as described previously (4, 5). To distinguish between the inverted terminal repeats (ITRs), two libraries, each comprising a pool of PCR amplicons corresponding to half of the *Capripoxvirus* genome, were prepared using a KAPA HyperPrep kit (Roche). MiSeq sequencing (reagent kit v3 with 2 × 300-bp paired-end sequencing; Illumina) was performed by the Genomics Core (Leuven, Belgium) and generated 1,918,474 and 2,187,236 paired-end reads per library (mean read length, 294 bp). The quality of the data was evaluated using FastQC v0.11.3 (https://www.bioinformatics.babraham.ac.uk/projects/fastqc/), and Trim Galore! v0.3.8 (https://www.bioinformatics.babraham.ac.uk/projects/trim_galore/) was used for read trimming based on quality (Q score, >30) and length (>80 bp; 5′ clip for R1 and R2 = 20). For each library, a subset of 20,000 trimmed paired-end reads were assembled *de novo* into a single contig (mean coverage, 85×) using SPAdes v3.9.0 with k values of 21, 33, and 55 (6). The contigs from both libraries were manually merged into a sequence of 150,719 bp, with an evenly distributed average G+C content of 25.98%. The LSDV 210LSD-249/BUL/16 is characterized by a 145,947-bp central coding region flanked by two ITRs of at least 2,386 bp and contains all expected LSDV open reading frames (ORFs). NCBI BLAST analysis (7) showed that 210LSD-249/BUL/16 shares 99.99% nucleotide identity with the contemporary LSDV field isolates from Israel (2012; GenBank accession number KX894508), Greece (2015; accession number KY829023), and Kazakhstan (2016, accession number MN642592). Annotation and amino-acid gene prediction was performed using GATU software (downloaded from https://4virology.net/virology-ca-tools/gatu/) (8) relative to the LSDV field isolate Evros/GR/15 (accession number KY829023), coupled with Vgas (3, 9), and discrepancies were confirmed by Sanger sequencing. A single nucleotide mutation and 2 single nucleotide indels were identified. The localization of the nucleotide modifications and their impact on the coding sequence are given in Table 1. These findings confirm that contemporary LSDV field strain genome sequences can be distinguished by only a couple of mutations. As a consequence, only (near-)complete genome sequences of circulating LSDV strains will allow accurate outbreak tracing.

**TABLE 1:** Nucleotide modifications and their impact on the coding sequence of LSDV field strain 210LSD-249/BUL/16 compared to LSDV field strain Evros/GR/15^*a*^

Gene or IR^*b*^	Nucleotide modification^*c*^	Impact on coding sequence	Coverage depth at position (×)	Mutation confirmed by Sanger sequencing
LD26b	D	Premature stop: 324 → 201 AA^*d*^	8,815	9T → 8T
LD076	M	Asp → Asn	7,947	G → A
IR LD146–LD147	I		6,185	13A → 14A

a GenBank accession number for LSDV field strain Evros/GR/15, KY829023.

b IR, intergenic region.

c M, mutation; D, deletion; I, insertion.

d AA, amino acids.

### Data availability.

The LSDV 210LSD-249/BUL/16 sequence has been deposited in GenBank under accession number MT643825, and the raw data have been submitted to the SRA under BioProject number PRJNA641001.
